# The Mini-Incision Technique Versus Conventional Open Approach for Carpal Tunnel Release: A Retrospective, Comparative Cohort Study

**DOI:** 10.7759/cureus.47814

**Published:** 2023-10-27

**Authors:** Saran Malisorn

**Affiliations:** 1 Orthopaedics, Naresuan University, Phitsanulok, THA

**Keywords:** satisfactory surgical outcomes, quickdash score, open release, mini-incision, carpal tunnel syndrome

## Abstract

Background and objective

Open carpal tunnel release (OCTR) is the gold standard technique for treating carpal tunnel syndrome (CTS). While mini-incision carpal tunnel release (MCTR) has been introduced as an alternative approach, there have been some concerns over its effectiveness and risks. In light of this, the aim of the study was to compare the long-standing clinical outcomes after MCTR with those following OCTR.

Methods

We employed a retrospective cohort design for this study. Patients were classified into two surgery groups, followed up for two years, and compared in terms of the following variables: duration of symptoms; pinch strength; grip strength; 2-point discrimination; visual analog scale (VAS) score; Levine symptom score; Levine function score; quick disabilities of the arm, shoulder, and hand (QuickDASH) score; wound pain; and pillar pain.

Results

The study included data regarding 120 patients, 71.66% of whom were females. The MCTR and OCTR groups were similar in terms of age, sex, duration of symptoms, and certain other aspects at baseline. The operation duration (15.15 ±2.20 vs. 25.01 ±2.15 minutes, p<0.01) and incision length (11.425 ±1.56 vs. 20.35 ±2.43 mm, p<0.01) were significantly shorter in the MCTR group compared to the OCTR group. Wound pain and pillar pain were not documented in the MCTR group at three and six months. The OCTR group had pillar pain in 25% of the patients till two years postoperatively.

Conclusion

Based on our findings, we propose that MCTR is superior to OCTR. The mini-incision technique has the advantages of small incision and scar, low pain, and faster recovery. Moreover, the technique was also found to be safe with no major complications or recurrence of symptoms. Further randomized control trials may help to re-evaluate the technique and validate our findings.

## Introduction

Carpal tunnel syndrome (CTS) is a common upper limb neuropathy due to the compression of the median nerve at the fibro-osseous canal, the carpal tunnel situated in the volar wrist. The cause of CTS is predominantly structural, and individual components as well as environmental and occupational factors have been implicated [1,2]. This condition is characterized by intermittent numbness, tingling, and pain in the fingers and palm, causing problems in daily activities and thereby decreasing productivity and the quality of life [3]. Nonsurgical methods such as wrist splints, steroid injections, and therapeutic exercise are the treatment methods employed at the initial stage, followed by surgery that involves the division of the transverse carpal ligament and releasing the stress in the median nerve. While the surgical technique called open carpal tunnel release (OCTR) has been practiced for a long time, approaches such as mini-incision carpal tunnel release (MCTR) and endoscopy-assisted carpal tunnel release (ECTR) are gaining prominence these days [4]. The major goals for patients undergoing these surgical methods are symptom relief, faster recovery, cosmetic satisfaction, and procedure effectiveness.

OCTR has been the first-line technique of choice since it was first described by Phalen in 1966 [5], but it involves a long and curvilinear palmar incision. Notably, every method has its own pros and cons; for instance, while conventional OCTR is associated with a high success rate, it results in a large scar, and patients who undergo this procedure take a longer time to recover [6,7]. On the other hand, ECTR is associated with less pain and early recovery but there have been concerns about the possibility of nerve injury [8]. Therefore, a method that provides better outcomes in a short time, which is also cost-effective and associated with fewer complications could be of huge benefit to this patient population. MCTR, which involves a small surgical incision of 1.5 cm compared to 3.5 cm in OCTR, has been introduced as an alternative approach that leads to better outcomes in a shorter time for carpal tunnel release (CTR). In this study, the long-term clinical outcomes of patients who underwent OCTR and MCTR are compared. To our knowledge, there is a dearth of studies that involve a matched-pair analysis of MCTR and OCTR. We hypothesize that MCTR is better for median nerve decompression than the OCTR method.

## Materials and methods

Study design and population

A retrospective cohort study was conducted among CTS patients who underwent either OCTR or MCTR surgery at Naresuan University Hospital between January 3, 2018, and December 30, 2020, and whose trigger finger symptoms were treated with open surgery or percutaneous surgery of the A1 pulley. Adults aged more than or equal to 18 years and diagnosed with CTS based on symptoms and electromyography were included in the study. The exclusion criteria were as follows: those who had undergone earlier management with steroid injection, or surgery of the hand or fingers, gout, arthritis, and the presence of diabetes mellitus. Also, patients who were allergic to nonsteroidal anti-inflammatory drugs, and those with a history of chronic liver or biliary disease, gastric ulcers or gastrointestinal bleeding, asthma, and renal disease were excluded from the study. This study adhered to the tenets of the Declaration of Helsinki and was approved by the ethical committee of Naresuan University (P3-0059/2566), (COA No.166/2023).

Sample size

The sample size was estimated by comparing two proportions (two-tailed test). The proportions p1=0.05 (mini-incision approach) and p2=0.23% (conventional open approach) were adopted based on a previous study [9]. Alpha-type error and statistical power were set at 5% and 80%, respectively. The estimated sample size was 58 per group (N=116).

Surgical procedure

The surgery for CTS was performed in the outpatient department of the Naresuan University Teaching Hospital. The patients were positioned supine with their arms stabilized on the surgery table using a tourniquet. A standard aseptic surgery technique was employed and 2 ml of 1% plain lidocaine hydrochloride was injected as local anesthetics. In OCTR, a longitudinal incision of about 3.5 cm was made alongside the median palmar crease up to 0.5 cm distal to the wrist crease. Next, the subcutaneous tissue was dismembered, and the transverse carpal ligament was sectioned starting at the ulnar border towards the proximal direction. The median nerve was recognized and any damage was avoided to it during the procedure. In MCTR, a 1-1.5 cm incision was made proximally at the crossing point between Kaplan’s cardinal line and a line sketched along the radial border of the third web. The subcutaneous tissue was cleared and the transverse carpal ligament was tunneled in the proximal direction using a dilator. Next, a protective guide was inserted to protect the palmar arteries and median nerves when the transverse carpal ligament was dissected with a special surgical knife. In the end, the wound was cleaned, stitched, and dressed to protect the underlying structures and to allow for soft tissue healing. The patients were prescribed analgesics and antibiotics after both surgeries. They were scheduled for follow-up visits at two weeks (when stitches were removed), and one, three, six, 12, and 24 months to assess the wound, postoperative pain, complications, symptoms, and functional outcomes.

OCTR is a surgical procedure performed to alleviate the symptoms of CTS. CTS is a medical condition characterized by the compression of the median nerve in the wrist, resulting in symptoms such as numbness, tingling, pain, and weakness in the hand and fingers.

Detailed Description of the OCTR Technique

An overview of the OCTR procedure is provided below:

Incision: in OCTR, the surgeon makes an incision typically in the palm of the hand, near the base of the wrist. This incision allows access to the carpal tunnel area length incision 2.5-3 cm.

Transverse carpal ligament release: the primary objective of the surgery is to release the transverse carpal ligament. This ligament forms the roof of the carpal tunnel, and when it is cut or released during the procedure, it relieves pressure on the median nerve.

Visualization and confirmation: surgeons identify and dissect the ligament while avoiding damage to other nearby structures.

Closure: once the ligament is adequately released, the incision is closed with sutures.

MCTR aims to minimize the wound size to 1.5 cm to attain a shorter recovery time and a better aesthetic appearance.

Step 1: identify the region of operation and the size of the operation - the surgeon identifies the incision surgery area, and the size of the wound is approximately 1.5 cm.

Step 2: a small incision around 1.5 cm; identify palmar fascia and transverse carpal ligament.

Step 3: insert a retractor to separate the transverse carpal ligament from the fat tissue layer. The elevated side of the retractor is inserted to see the transverse carpal ligament and cut with blade No. 11; visualize by loupe and protect the median nerve by surgical clamp while cutting the transverse carpal ligament. The length of the blade is 2.5 cm, which can be separated. The tip of the transverse carpal ligament is visually cut to avoid the median branch nerve. The proximal margin is also cut inside the palmaris longus tendon to avoid injury of the median nerve of the palmar skin as this nerve is always on the radial side of the palmaris longus tendon.

Step 4: stitch the wound size of 1.5 cm.

Detailed Description of the MCTR Technique

The MCTR technique was performed as follows:

1. Patient positioning: patients were placed in the supine position on the operating table with the affected arm positioned for easy access to the surgical site. 

2. Anesthesia: a local anesthetic, such as lidocaine, was administered to the wrist and surrounding area to ensure pain relief during the procedure. 

3. Incision: a (specific-length) transverse incision was made in the palmar aspect of the wrist, centered over the carpal tunnel. Care was taken to place the incision in a cosmetically favorable position while allowing sufficient access to the surgical field.

4. Protective guide: a protective guide, which is an essential component of the mini-incision technique, was employed to safeguard the adjacent structures, including the median nerve. The guide served to create a safe passage for the surgical instruments underneath the transverse carpal ligament while minimizing the risk of unintended injuries.

5. Ligament release: under the guidance of the protective guide, a minimally invasive release of the transverse carpal ligament was performed using specialized instruments. The release was executed carefully to ensure complete transection of the ligament.

6. Closure: after ligament release, the protective guide was removed, and the incision was meticulously closed by using appropriate suturing techniques to minimize tension and ensure an aesthetically pleasing result.

7. Postoperative care: patients were provided with postoperative care instructions, including wound care, pain management, and early mobilization exercises.

This detailed description provides a clear overview of the MCTR technique, emphasizing the use of the protective guide, which is a key element of the procedure. The inclusion of such details enhances the transparency and replicability of the study, facilitating a better understanding of the surgical approach used in the research.

Figures 1-8 illustrate the MCTR surgery procedure.

**Figure 1:**
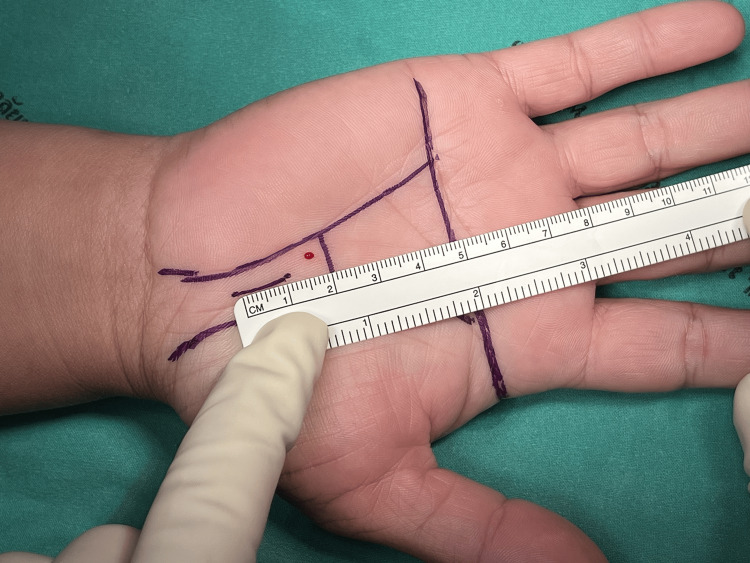
MCTR surgery picture - image 1 In MCTR, a 1-1.5 cm incision is made proximally at the crossing point between Kaplan’s cardinal line and a line sketched along the third web space MCTR: mini-incision carpal tunnel release

**Figure 2:**
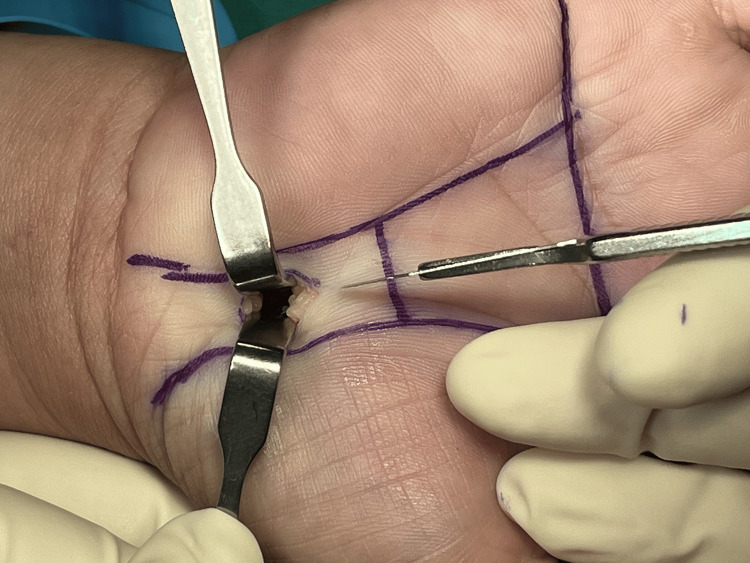
MCTR surgery picture - image 2 The subcutaneous tissue is cleared MCTR: mini-incision carpal tunnel release

**Figure 3:**
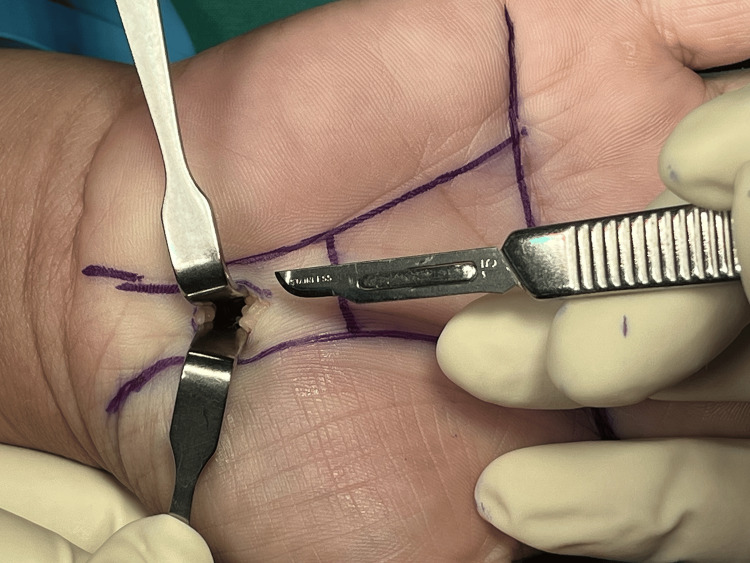
MCTR surgery picture - image 3 The transverse carpal ligament is tunneled in the proximal direction by using a dilator MCTR: mini-incision carpal tunnel release

**Figure 4:**
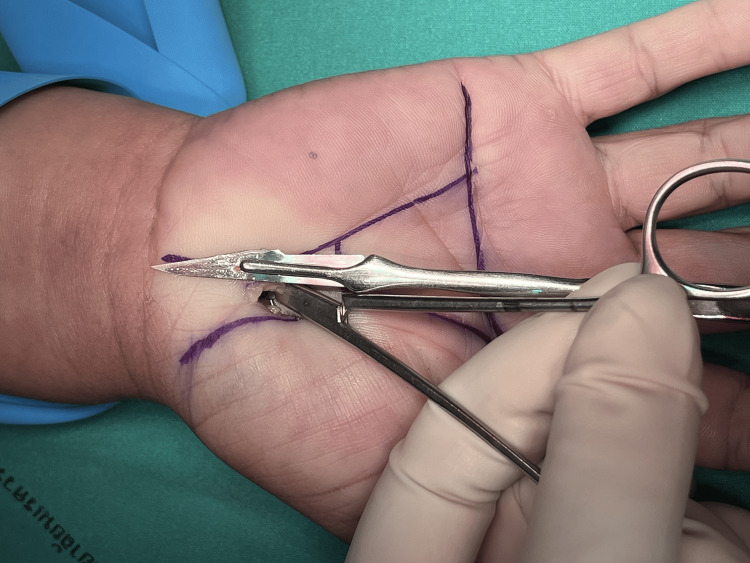
MCTR surgery picture - image 4 A protective guide is inserted to protect the palmar arteries and median nerves MCTR: mini-incision carpal tunnel release

**Figure 5:**
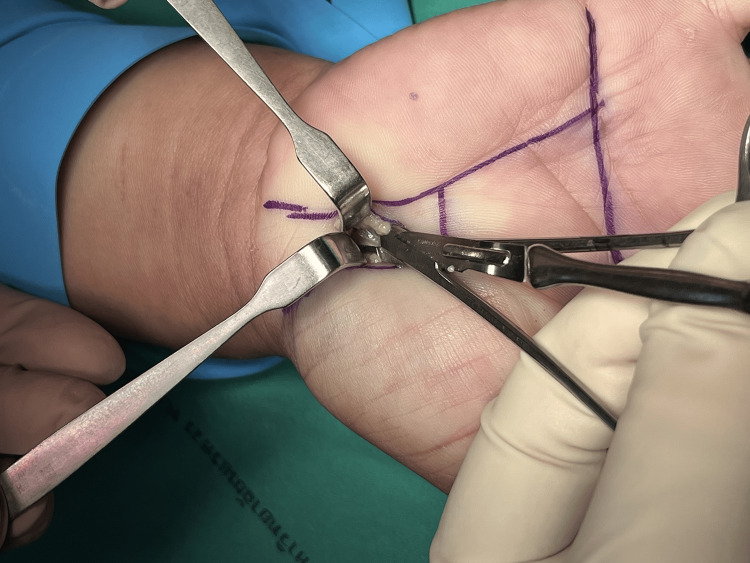
MCTR surgery picture - image 5 The transverse carpal ligament is dissected with a special surgical knife MCTR: mini-incision carpal tunnel release

**Figure 6:**
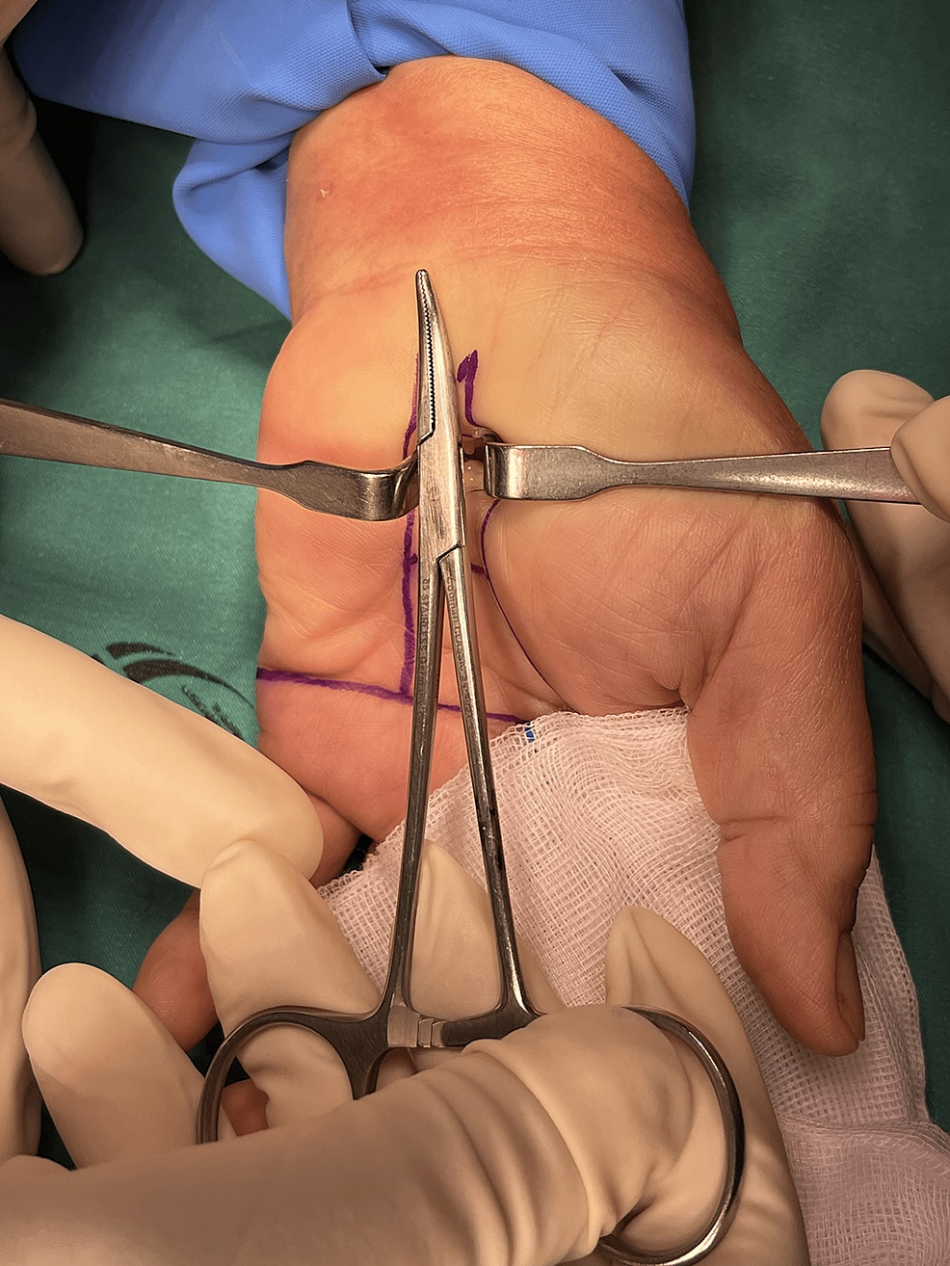
MCTR surgery picture - image 6 A protective guide, which is an essential component of the mini-incision technique, is employed to safeguard the adjacent structures, including the median nerve. The guide serves to create a safe passage for the surgical instruments underneath the transverse carpal ligament while minimizing the risk of unintended injuries MCTR: mini-incision carpal tunnel release

**Figure 7:**
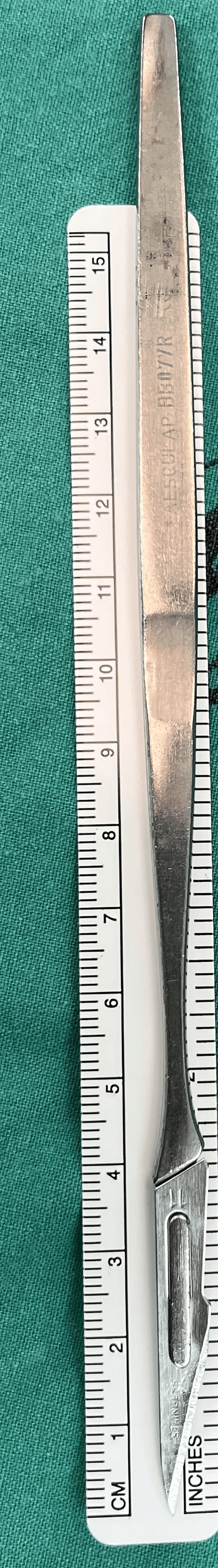
MCTR surgery picture - image 7 Under the guidance of the protective guide, a minimally invasive release of the transverse carpal ligament is performed by using a specialized knife MCTR: mini-incision carpal tunnel release

**Figure 8:**
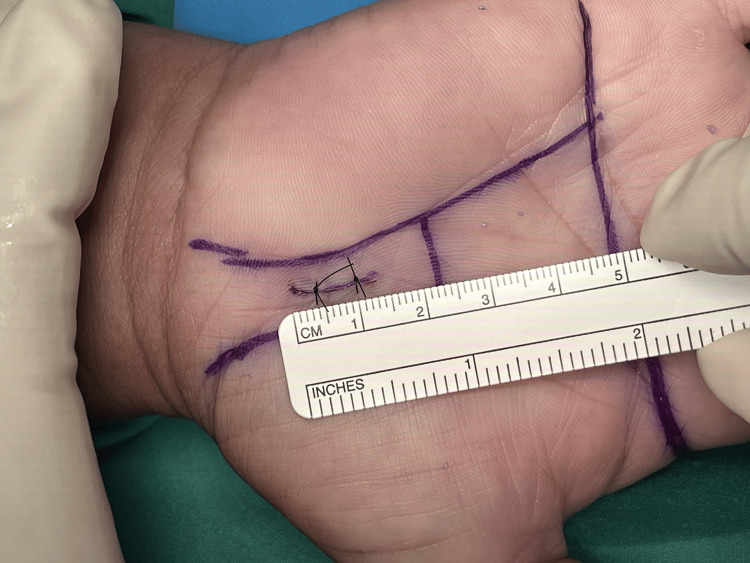
MCTR surgery picture - image 8 The wound is cleaned, stitched, and dressed to protect the underlying structures and to allow for soft tissue healing MCTR: mini-incision carpal tunnel release

Variables and data collection

The hospital’s computer system and patient’s medical records were investigated for gender, age, hand-side affected, duration of symptoms, pinch strength, grip strength, 2-point discrimination, visual analog scale (VAS) score, Levine symptom score, Levine function score, QuickDASH (disabilities of the arm, shoulder, and hand) score, wound pain, and pillar pain on each follow-up after surgery. Other details documented were operation duration and length of the incision. Pinch and grip strength was measured according to standard methods by using a pinch meter and dynamometer, respectively. In the 2-point discrimination test, the patients were asked to close their eyes, and the pressure was applied at two adjacent points in the index finger. VAS scale was used to assess pain while moving the wrist, which consists of a 10 cm line with one end designated as ‘no pain’ and the other end as ‘severe pain’. The Boston Carpal Tunnel Syndrome Questionnaire (BCTSQ) was used to score the symptom and function severity, respectively [10]. Each question included five possible replies with points assigned ranging from 1 (mild) to 5 (severe), and the total for each section was calculated as the average of the responses to the individual items. The Levine symptom scale measures pain, weakness, tingling, numbness, and difficulty with motor activities whereas the Levine function scale was used to score the daily life tasks. The QuickDASH questionnaire with 11 items, scored on a 0-5 Likert scale, was applied to measure physical disability and symptoms in the upper limb [11]. Wound pain was considered when located centrally underneath the surgery scar. Pillar pain was defined as discomfort at the thenar or hypothenar eminence or both while tightly gripping the hand.

Statistical analysis

Statistical analysis was performed by using IBM SPSS Statistics version 17 (IBM Corp., Armonk, NY). Descriptive data were presented as frequency, percentage, mean, and standard deviation (SD). Continuous variables in two groups were compared by the Student’s t-test and categorical variables were analyzed using the Chi-square test. A p-value <0.05 was considered statistically significant.

## Results

Baseline characteristics

Of the 120 patients [MCTR (n=60), OCTR (n=60)], the majority were female (71.66%). The patients in the MCTR and OCTR groups were statistically similar in terms of sex (p=0.99), age (p=0.91), and duration of symptoms (p=0.70). The average age of the participants was 55.48 ±5.42 years and 55.36 ±5.90 years in the OCTR and MCTR groups, respectively. There was no statistically significant difference with regard to the affected hand-side between the groups (p=0.85); however, right-side involvement was higher in both groups. Similarly, the groups were statistically similar in terms of pinch strength, grip strength, 2-point discrimination, and Levine function score at baseline. However, the VAS score (p=0.01), Levine symptom score (p<0.01), and QuickDASH score (p=0.02) were significantly higher in the OCTR group than in the MCTR group (Table 1).

**Table 1:** Baseline characteristics of the study participants MCTR: mini-invasive carpal tunnel release; OCTR: open carpal tunnel release; SD: standard deviation; VAS: visual analog scale; 2-PD: 2-point discrimination; DASH: disabilities of the arm, shoulder, and hand

Variables	MCTR (n=60)	OCTR (n=60)	P-value
Gender, n (%)			0.99
Male	17 (28.33)	17 (28.33)	
Female	43 (71.37)	43 (71.67)	
Age, years, mean ±SD	55.36 ±5.90	55.48 ±5.42	0.91
Affected side, n (%)			0.85
Left	23 (38.33)	24 (40.0)	
Right	37 (61.67)	36 (60.0)	
Duration of symptoms, months, mean ±SD	7.74 ±3.11	7.53 ±2.87	0.70
Pinch strength, kg, mean ±SD	5.25 ±1.15	5.18 ±1.31	0.72
Grip strength, kg, mean ±SD	14.49 ±2.52	13.74 ±2.41	0.09
2-PD, mm, mean ±SD	5.89 ±0.79	6.1 ±1.07	0.22
VAS score, mean ±SD	8.75 ±0.98	9.18 ±0.91	0.01
Levine symptom score, mean ±SD	4.24 ±0.34	4.43 ±0.37	<0.01
Levine function score, mean ±SD	4.32 ±0.35	4.45 ±0.38	0.06
QuickDASH score, mean ±SD	4.26 ±0.34	4.41 ±0.40	0.02

Clinical outcomes

The duration of operation in the OCTR group was significantly higher (p<0.01) than in the MCTR group (25.01 ±2.15 vs. 15.15 ±2.20 minutes). Similarly, the average incision length was statistically significantly (p<0.01) longer in the OCTR group (20.35 ±2.43 mm) compared to the MCTR group (11.425 ±1.56 mm). On post-surgery follow-up visits at two weeks, pinch strength, gripping strength, point discrimination value, and wound pain were not statistically significant between the groups, whereas the VAS score, Levine symptom score, Levine function score, QuickDASH score, and pillar pain were significantly higher in the OCTR group than the other study group. Next, at the one-month follow-up, the number of patients in the MCTR group having wound and pillar pain was statistically significantly lower than in the OCTR group. Other study parameters (except pinch and gripping strength) were significantly higher in the OCTR group compared to the other group (Table 2).

**Table 2:** Clinical outcomes at two-week and one-month follow-ups after the surgery *P-value <0.05 MCTR: mini-invasive carpal tunnel release; OCTR: open carpal tunnel release; SD: standard deviation; 2-PD: 2-point discrimination; VAS: visual analog scale; DASH: disabilities of the arm, shoulder, and hand

Variables	2 weeks			1 month		
	MCTR	OCTR	P-value	MCTR	OCTR	P-value
Pinch strength, kg, mean ±SD	5.40 ±1.12	5.38 ±1.19	0.91	6.25 ±1.08	6.15 ±1.25	0.65
Grip strength, kg, mean ±SD	14.70 ±2.81	13.94 ±2.35	0.1	16.18 ±2.90	15.39 ±2.45	0.11
2-PD, mm, mean ±SD	5.78 ±0.80	6.00 ±1.05	0.19	4.65 ±0.95*	5.10 ±1.07*	0.01
VAS score, mean ±SD	2.81 ±0.81*	3.71 ±1.00*	<0.01	1.65 ±0.68*	2.4 ±0.71*	<0.01
Levine symptom score, mean ±SD	3.80 ±0.48*	4.04 ±0.31*	<0.01	2.85 ±0.44*	3.29 ±0.41*	<0.01
Levine function score, mean ±SD	3.81 ±0.47*	4.06 ±0.32*	<0.01	2.85 ±0.44*	3.33 ±0.43*	<0.01
QuickDASH score, mean ±SD	3.77 ±0.47*	4.01 ±0.35*	<0.01	2.81 ±0.49	3.24 ±0.43*	<0.01
Wound pain, n (%)	58 (96.67)	59 (98.33)	0.55	1 (1.67)*	59 (98.33)*	<0.01
Pillar pain, n (%)	56 (93.33)*	60 (100)*	0.04	20 (33.33)*	60 (100)*	<0.01

At the three-month and six-month post-surgery follow-up visits, the patients in the OCTR group had a significantly higher 2-point discrimination, VAS score, Levine symptom score, Levine function score, and QuickDASH score than the MCTR group. However, pinch and gripping strength were similar between the groups. The incidence of wound pain and pillar pain was significantly lower in the MCTR group than in the OCTR group. At three months, none of the patients reported wound pain and 5% had pillar pain after the MCTR compared to 35% wound pain and 93.33% pillar pain in the OCTR group. At six months, neither wound pain nor pillar pain was documented in the MCTR group but recorded in the conventional OCTR group, and 88.33% of the patients in the OCTR had pillar pain, as shown in Table 3.

**Table 3:** Clinical outcomes at three- and six-month follow-ups after the surgery *P-value <0.05 MCTR: mini-invasive carpal tunnel release; OCTR: open carpal tunnel release; SD: standard deviation; 2-PD: 2-point discrimination; VAS: visual analog scale; DASH: disabilities of the arm, shoulder, and hand

Variables	3 months			6 months		
	MCTR	OCTR	P-value	MCTR	OCTR	P-value
Pinch strength, kg, mean ±SD	7.04 ±1.05	6.95 ±1.30	0.66	7.83 ±0.98	7.67 ±1.27	0.43
Grip strength, kg, mean ±SD	17.64 ±2.75	16.92 ±2.48	0.13	19.04 ±2.43	18.35 ±2.50	0.13
2-PD, mm, mean ±SD	3.93 ±0.75*	4.26 ±1.01*	0.04	3.35 ±0.50	4.29 ±5.39	0.18
VAS score, mean ±SD	0.65 ±0.60*	1.3 ±0.64*	<0.01	0.05 ±0.21*	0.31 ±0.50*	<0.01
Levine symptom score, mean ±SD	1.93 ±0.49*	2.47 ±0.49*	<0.01	1.11 ±0.32*	1.57 ±0.56*	<0.01
Levine function score, mean ±SD	1.89 ±0.45*	2.49 ±0.49*	<0.01	1.09 ±0.28	1.57 ±0.52	<0.01
QuickDASH score, mean ±SD	1.87 ±0.47*	2.41 ±0.51*	<0.01	1.10 ±0.30*	1.52 ±0.51*	<0.01
Wound pain, n (%)	0 (0)*	21 (35.0)*	<0.01	0 (0)	1 (1.67)	0.31
Pillar pain, n (%)	3 (5.0)*	56 (93.33)*	<0.01	0 (0)*	53 (88.33)*	<0.01

Table 4 shows the clinical outcomes at the one- and two-year follow-ups after the surgery. At one year, the variables, i,e., point discrimination, Levine symptom score, Levine function score, Quick DASH score, and pillar pain were significantly higher in the OCTR group than in the MCTR group. None of the patients in either group had wound pain. At two years, except for the number of patients with pillar pain, which was statistically higher in the OCTR group (25%) than the MCTR group (0%), there were no statistically significant differences between the two groups with regard to other study variables. None of the patients in either group were reported to have wound infection and reoperation to alleviate symptoms in the study period.

**Table 4:** Clinical outcomes at one- and two-year follow-ups after the surgery *P-value <0.05 MCTR: mini-invasive carpal tunnel release; OCTR: open carpal tunnel release; SD: standard deviation; 2-PD: 2-point discrimination; VAS: visual analog scale; DASH: disabilities of the arm, shoulder, and hand

Variables	1 year			2 years		
	MCTR	OCTR	P-value	MCTR	OCTR	P-value
Pinch strength, kg, mean ±SD	8.54 ±0.96	8.39 ±1.26	0.45	9.25 ±1.14	9.10 ±1.32	0.50
Grip strength, kg, mean ±SD	20.41 ±2.21	19.81 ±2.33	0.15	21.9 ±1.82	21.42 ±2.19	0.20
2-PD, mm, mean ±SD	2.94 ±0.36*	3.17 ±0.51*	<0.01	2.66 ±0.46	2.85 ±0.51	0.08
VAS score, mean ±SD	0 ±0	0.16 ±0.12	0.31	0	0	NA
Levine symptom score, mean ±SD	1 ±0*	1.14 ±0.31*	<0.01	1 ±0	1 ±0	NA
Levine function score, mean ±SD	1 ±0*	1.12 ±0.29*	<0.01	1 ±0	1 ±0	NA
QuickDASH score, mean ±SD	1 ±0*	1.12 ±0.31*	<0.01	1 ±0	1 ±0	NA
Wound pain, n (%)	0	0	NA	0	0	NA
Pillar pain, n (%)	0 (0)*	49 (81.67)*	<0.01	0 (0)*	15 (25.0)*	<0.01

**Figure 9:**
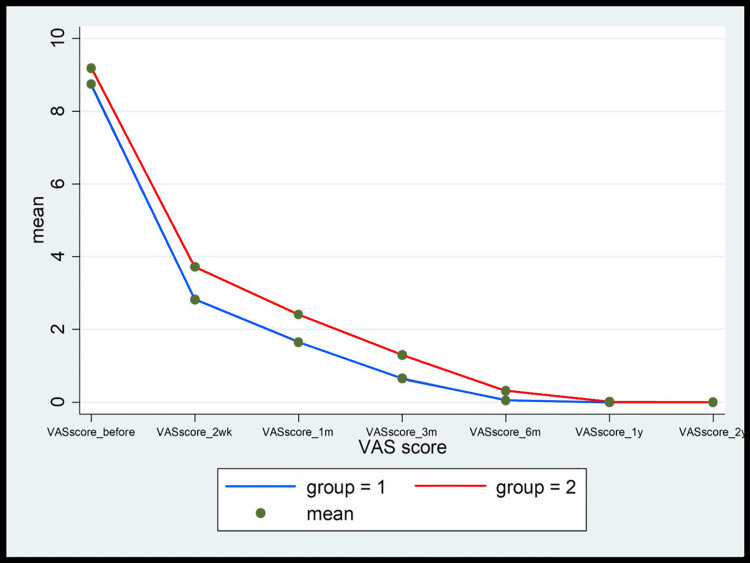
The comparison of the VAS score between MCTR (group 1) and OCTR (group 2) patients during the follow-up at 2 weeks and 1, 3, 6, 12, and 24 months MCTR: mini-invasive carpal tunnel release; OCTR: open carpal tunnel release; VAS: visual analog scale

**Figure 10:**
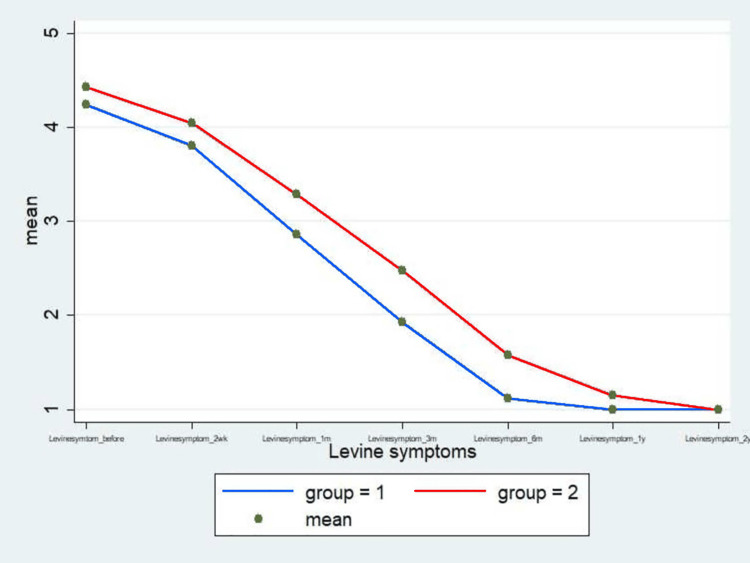
The comparison of the Levine symptom score between MCTR (group 1) and OCTR (group 2) patients during the follow-up at 2 weeks and 1, 3, 6, 12, and 24 months MCTR: mini-invasive carpal tunnel release; OCTR: open carpal tunnel release

**Figure 11:**
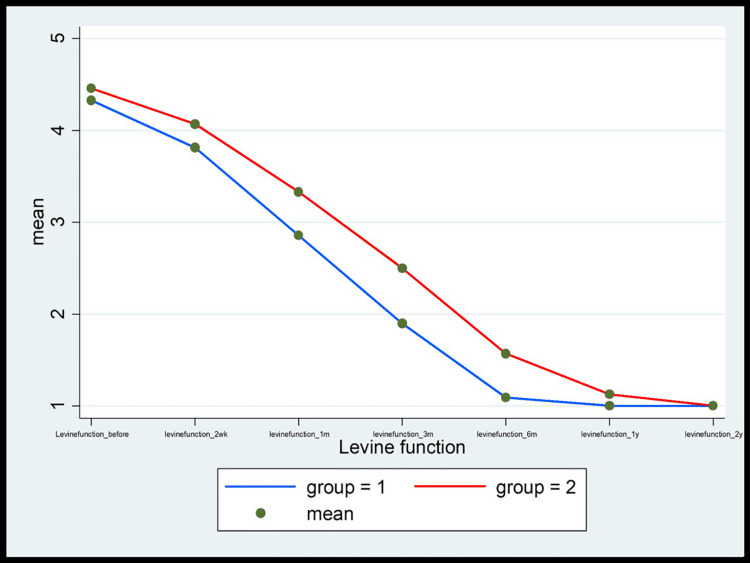
The comparison of the Levine function score between MCTR (group 1) and OCTR (group 2) patients during the follow-up at 2 weeks and 1, 3, 6, 12, and 24 months MCTR: mini-invasive carpal tunnel release; OCTR: open carpal tunnel release

**Figure 12:**
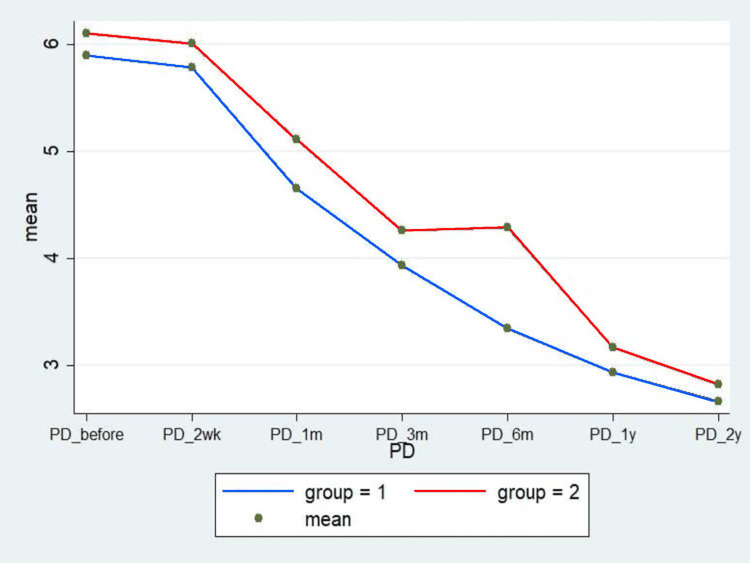
The comparison of the 2-point discrimination score between MCTR (group 1) and OCTR (group 2) patients during the follow-up at 2 weeks and 1, 3, 6, 12, and 24 months MCTR: mini-invasive carpal tunnel release; OCTR: open carpal tunnel release

**Figure 13:**
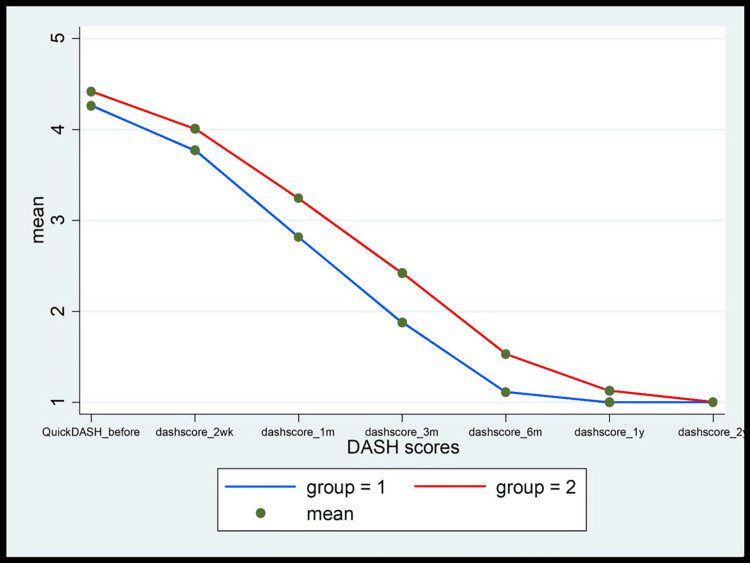
The comparison of the QuickDASH score between MCTR (group 1) and OCTR (group 2) patients during the follow-up at 2 weeks and 1, 3, 6, 12, and 24 months MCTR: mini-invasive carpal tunnel release; OCTR: open carpal tunnel release; DASH: disabilities of the arm, shoulder, and hand

Figures 9-13 show the comparative analysis between the two groups in terms of the study variables during the follow-ups.

## Discussion

In this study, we aimed to investigate the clinical outcomes during follow-up periods spanning two years in CTS patients who underwent either MCTR or OCTR. The major highlight of the current study is that it involved matched populations in terms of number, age, gender, affected hand, duration of symptoms, and other aspects. Our findings revealed that MCTR is superior to conventional OCTR given the short duration of wound and pillar pain and early relief of the symptoms associated with the former. Thus, the study emphasizes that MCTR is an ideal approach for the treatment of CTS. The average age of the patients in the study was 55 years and it included more females than males. This is in line with a previous study, which reported that CTS is common in the age groups of late 40s and 50s and higher in females [12]. This association with the female sex is probably due to hormonal changes, especially during pregnancy or menopause [13]. One study has revealed that CTS is higher in pregnant than in non-pregnant women [14]. Although demographics and other variables were similar between the groups at the baseline, the VAS score, Levine symptom score, and QuickDASH score were significantly higher in the OCTR group than in the other study group. Given the nature of the study and large datasets, it was nearly impossible to match all the information between the study groups. Nevertheless, the average duration of symptoms was also statistically similar (p=0.70) between the groups. The symptom duration in our study was about 7.6 months, which is comparable to a previous study reporting 6.5 months [9].

One of the major advantages observed in the MCTR group in the study was the short surgery duration and the smaller incision length compared to the other technique. The surgery duration in the study was statistically significantly different between the groups, which aligns with the findings of one earlier study [15] but contrasts with those of another study [9]. The size of the incision is important given the scar formation during the healing process. A scar is not only critical from a cosmetic viewpoint but, in some cases, it reforms a pseudo-transverse carpal ligament or scarring of the median nerve [16,17], thereby leading to the reoccurrence of symptoms. Reduced scar formation on open mini-skin incision surgery for median nerve release has been described in a previous study [18]. On the other hand, some studies have reported OCTR increasing the incidence of scar formation [19,20].

Another important finding in the MCTR group in the study was the "incisional" pain, which subsequently subsided within one month of the surgery (1.67% vs. 98.33% in the OCTR group). Likewise, pillar pain also decreased in a significant proportion of patients within one month of the surgery in the MCTR group compared to the OCTR group. The wound pain completely disappeared in three months and the pillar pain resolved by six months in the MCTR group. However, pillar pain was recorded in 25% of the patients in the OCTR group for the entire duration of follow-up visits (two years), endorsing the earlier published findings that the OCTR increases the incidence of postoperative pillar pain [20]. Moreover, an overall comparison of the VAS score (Figure 9), Levine symptom score (Figure 10), Levine function score (Figure 11), 2-point discrimination measure (Figure 12), and QuickDASH score (Figure 13) among the patients in both groups at baseline and post-surgery follow-up revealed that the MCTR group outperformed the OCTR group. An early decrease in the QuickDASH score and symptom and function scores after the surgery including shorter duration and lower severity of wound and pillar pain indicated that MCTR provides better and faster relief of the symptoms. This is vital given that it enables patients' early return to work and an improvement in their quality of life. The results are similar to those of Bai et al. in terms of incision length and Levine score [9] and to those of Schwarz et al. in terms of scar sensibility [15].

Pinch and grip strength was not significantly different between the groups but improved over post-surgery follow-up. No complications or incomplete release of the transverse carpal ligament was recorded in both methods, indicating that MCTR is safe and reliable. There are some limitations to this study. This was a retrospective study conducted at a single center, which may have affected the findings. The study compared data between the OCTR and MCTR groups, but the inclusion of other techniques such as ECTR could have provided a much clearer comparison and more insightful conclusions. Despite the limitations, our study has provided strong evidence by comparing findings over two years of follow-up instead of long-term clinical outcomes in the group-matched patients.

Analysis of the relationship between symptoms, severity, and surgical technique

Exploring the potential relationship between the duration and severity of CTS symptoms and the outcomes of the surgical techniques is important. We recognize that these factors can play a critical role in determining the efficacy of the chosen approach. To this end, we conducted a subgroup analysis to assess the impact of symptom duration and severity on the results.

Duration of Symptoms

Patients were categorized into subgroups based on the duration of their CTS symptoms: short-duration (less than six months), medium-duration (6-12 months), and long-duration (over 12 months) groups. This allowed us to explore whether the outcomes of the surgical techniques varied based on the severity of symptoms.

Symptom Severity

The severity of CTS symptoms was assessed using a validated scoring system: the Levine symptom score. Data analysis revealed trends for much better results and indicated a potential relationship between symptom duration, severity, and the results of the surgical techniques.

Based on our findings, MCTR is a safe and effective surgical technique for the management of severe CTS (high Levine score). The results indicate that the Levine symptom score, Levine function score, QuickDASH score, and VAS score significantly improved at all follow-up periods, supporting the use of MCTR as a viable treatment option in cases of advanced CTS.

## Conclusions

The current study revealed that MCTR is superior to OCTR for carpal tunnel release. The mini-incision technique has the advantage of small incision and scar, low pain, and faster recovery. Moreover, the technique was also found to be safe with no major complications or recurrence of symptoms. The mini-incision requires skilled and attentive surgeons, which means that it could prove beneficial for medical students and interns to study and practice it thoroughly in order to gain expertise and obtain patient satisfaction. We recommend that randomized control trials be conducted on this topic to re-evaluate the technique and validate our findings.
